# Environmental Lead Exposure and Glucose Dysregulation: A Critical Integrative Review of Epidemiological and Mechanistic Evidence

**DOI:** 10.3390/jox16060181

**Published:** 2026-09-24

**Authors:** Rolando Adair Facio Campos, Edgar Héctor Olivas Calderón, Cristo Omar Puente Valenzuela, Jorge Alejandro Sosa Gutiérrez

**Affiliations:** 1 Facultad de Ciencias Químicas Gómez Palacio, Universidad Juárez del Estado de Durango, Artículo 123 s/n, Fracc. Filadelfia, Gómez Palacio 35010, Durango, Mexico; rolando.facio@ujed.mx (R.A.F.C.); edgar.olivas@ujed.mx (E.H.O.C.); 2 Facultad de Ciencias Biológicas, Universidad Juárez del Estado de Durango, Universidad S/N, Núcleo Universitario, Fracc. Filadelfia, Gómez Palacio 35010, Durango, Mexico; puvacrom@ujed.mx

**Keywords:** type 2 diabetes mellitus, insulin resistance, pancreatic β-cell, hepatic gluconeogenesis, oxidative stress, toxicokinetics, coexposure, biomonitoring

## Abstract

Environmental lead (Pb) exposure has been proposed as a contributor to glucose dysregulation and type 2 diabetes mellitus (T2DM), but epidemiological consistency, causal interpretation, and human relevance of experimental models remain uncertain. Here, ‘integrative’ denotes an explicit cross-stream synthesis of human epidemiology, Pb-specific experimental toxicology, and adjacent mechanistic biology under common appraisal dimensions; it does not denote systematic-review status or formal evidence grading. Human findings are mixed and are dominated by cross-sectional studies using a single blood-Pb measurement. Positive associations are more frequent for triglyceride-glucose or other surrogate indices than for incident T2DM, whereas null estimates may reflect either absence of an effect or attenuation from exposure misclassification, restricted contemporary exposure ranges, and insufficient power. Experimental studies directly support impaired β-cell bioenergetics and insulin secretion and increased hepatic gluconeogenesis, but nominal doses, coadministration of a high-fat diet, and incomplete internal-dose reporting limit environmental extrapolation. Pb-specific support for Nrf2-mediated metabolic effects, TXNIP/NLRP3 activation in metabolic tissues, and gut-microbiota mediation remains indirect or preliminary. Real-world coexposure to other metals, particulate matter, and social stressors further limits lead-specific attribution. Overall, Pb is biologically plausible as one contributor to glucose dysregulation under some exposure and susceptibility conditions, but current evidence does not establish an independent causal effect on human T2DM. Prospective, mixture-aware studies with repeated and cumulative Pb biomarkers, adjudicated outcomes, and toxicokinetically anchored experimental designs are needed.

## 1. Introduction

Type 2 diabetes mellitus (T2DM) arises from the interaction of insulin resistance, progressive pancreatic β-cell failure, adipose-tissue dysfunction, hepatic glucose overproduction, and genetic, behavioral, and social determinants [1,2,3]. Environmental contaminants may add to this susceptibility, but mixture effects, correlated exposures, and common social causes make attribution to one toxicant difficult [4,5,6].

Lead (Pb) remains globally relevant because legacy contamination, mining and smelting, battery manufacture and recycling, informal industry, lead-glazed ceramics, paint, dust, water-distribution systems, and occupational settings continue to expose populations [7,8,9,10]. Pb has no established physiological function. It can compete with essential cations, bind protein thiols, disturb calcium-dependent signaling, impair mitochondria, and alter redox and epigenetic regulation [11,12,13,14,15]. These actions are compatible with impaired insulin secretion and action, but mechanistic compatibility is not equivalent to proof that environmental Pb independently causes T2DM.

Previous reviews have summarized the broad association between Pb and diabetes, relationships between multiple metals and T2DM, or general molecular pathways of heavy-metal toxicity [16,17,18]. The present article does not claim systematic-review status or unbiased molecular discovery. Its distinct contribution is a focused audit of three inferential gaps that are often collapsed in broader syntheses: (i) clinically diagnosed or incident T2DM versus surrogate endpoints such as HOMA-IR, TyG, fasting glucose, and HbA1c; (ii) effects directly demonstrated after Pb exposure in a metabolically relevant tissue versus mechanisms imported from general oxidative-stress or diabetes biology; and (iii) nominal experimental dose versus measured human internal exposure. This structure permits conclusions to be calibrated to what each evidence stream can actually establish.

Accordingly, this review contributes a study-level, direction-aware appraisal of the human literature; a dose-context table that separates administered exposure from achieved internal dose; a dedicated treatment of coexposure and mixture toxicology; and a mechanistic directness matrix. It deliberately does not use a literature-seeded protein-association analysis, because enrichment of redox and insulin pathways from a pathway-selected seed list cannot provide independent validation or novel pathway discovery.

Terminology is used operationally throughout. Glucose dysregulation is the umbrella term for any disturbance in glucose control. Dysglycemia denotes a measured abnormality in glycemia, such as fasting glucose, glucose tolerance, or HbA1c. Insulin resistance is used only when directly measured or estimated by a named index; TyG and HOMA-IR are identified as surrogates rather than clinical diabetes. Metabolic dysfunction is broader and may include lipid, hepatic, adipose, or endocrine abnormalities without dysglycemia. Diabetogenicity is reserved for evidence supporting progression toward diabetes, and T2DM refers to a clinically defined or adjudicated disease endpoint.

In this article, integrative denotes the explicit juxtaposition of four evidence streams—human epidemiology, metabolic experiments, Pb-specific redox toxicology, and adjacent diabetes biology—under common questions of temporality, exposure validity, endpoint directness, dose relevance, coexposure, and replication. The mechanistic synthesis is a conceptual linkage of molecular, cellular, organ, and population-level events; it does not constitute an unbiased omics network, STRING analysis, or independent causal validation. ‘Critical’ refers to symmetrical appraisal of supportive and null findings and explicit identification of untested inferential steps.

## 2. Review Methodology

### 2.1. Search Strategy and Eligibility

A targeted, author-led search was conducted in PubMed/MEDLINE and Europe PMC from database inception to 4 August 2026. Three predefined search families covered (i) epidemiological and metabolic outcomes, (ii) redox and organ-level mechanisms, and (iii) acute-dose and LD_50_ context. Database-specific strings, dates, and raw yields are reproduced in Appendix A. Searches were supplemented by backward and forward citation tracing of included primary studies and relevant reviews.

To broaden disciplinary coverage, a supplementary OpenAlex search was run on 6 September 2026 using 25 targeted title-and-abstract queries (12 metabolic, 10 mechanistic, and 3 acute-hazard queries) and a publication cutoff of 4 August 2026 [19]. Across PubMed, Europe PMC, and OpenAlex, the searches returned 6979 raw query hits (2281, 2387, and 2311, respectively). Overlapping OpenAlex query results were first consolidated, leaving 6597 records for cross-source and cross-stream deduplication. Matching by PMID, DOI, and normalized title yielded 3276 unique records; 3703 duplicate query occurrences were removed relative to the raw total. OpenAlex contributed 881 unique records not matched in PubMed or Europe PMC.

The original author-led review did not retain a contemporaneous item-level screening ledger. Consequently, numbers screened at title/abstract level, assessed in full text, and excluded by reason cannot be reconstructed reliably without false precision. We therefore do not present a PRISMA diagram or retrospective stage counts. Instead, Figure 1 reports the exact verifiable retrieval and deduplication counts, marks the unavailable screening stages explicitly, and provides an auditable inventory of the retained evidence. Claims that the selection process itself was ‘structured’ or ‘reproducible’ have been removed.

The final bibliography contains 106 retained sources, classified for source-accounting purposes into 24 human metabolic studies, 9 Pb-metabolic experiments, 7 Pb-specific redox/toxicology studies, 11 Pb-specific endocrine or lipid studies, 3 authoritative acute-hazard sources, 1 database-method source, and 51 contextual reviews or adjacent-biological sources. The human evidence table was expanded from 9 to 24 studies after the multidisciplinary search; this inventory is a transparent description of the retained corpus, not a claim of systematic completeness.

Eligible human studies measured Pb in blood, urine, bone, environmental media, or a defined occupational setting and reported clinically defined diabetes, fasting glucose, HbA1c, insulin, HOMA-IR, TyG, metabolic syndrome, or another directly relevant metabolic endpoint. Eligible experimental studies exposed mammalian cells or animals to a stated Pb compound and reported a glucose-regulatory, β-cell, hepatic, neural-insulin, redox, inflammatory, or gut–metabolic outcome. General redox or diabetes literature was retained only to identify explicitly labeled mechanistic bridges; it was not treated as direct Pb–metabolic evidence.

Main reasons for exclusion at full-text review were: absence of a Pb-specific exposure estimate or comparator; absence of a glucose-regulatory or directly relevant metabolic endpoint; a non-mammalian model without direct relevance to the review question; an antioxidant or therapeutic study without a Pb-only comparator; duplicate or less complete reporting from the same population; and a conference abstract or preprint without an eligible full report. These categories describe the applied eligibility logic; reason-specific counts were not retained.

Titles and abstracts were assessed for direct relevance, followed by full-text examination of exposure characterization, outcomes, and interpretability. Duplicate reports from the same population were consolidated by prioritizing the most complete analysis. Study inclusion and interpretation were performed by the author team, but independent duplicate screening was not undertaken. No protocol was registered, no PRISMA workflow was used, and the search was not designed to support a pooled effect estimate.

Bibliographic deduplication used PMID first, then DOI, and finally a normalized title after case folding and punctuation/spacing removal. This process addressed duplicate database records; it does not substitute for independent duplicate screening, which was not performed.

### 2.2. Evidence Extraction and Critical Appraisal

For each human study, appraisal considered six prespecified domains: design and temporality; exposure ascertainment, including timing, repetition, and cumulative relevance; outcome validity and distinction between clinical T2DM and a surrogate endpoint; control of demographic, clinical, socioeconomic, and coexposure confounding; precision and internal consistency; and plausible direction of reverse causation or measurement error. The appraisal records these considerations at study level and indicates how limitations could bias an estimate toward or away from the null.

To make the appraisal transparent at study level, each human study is assigned a concern profile for six domains: T, temporality; E, exposure ascertainment; O, outcome validity; C, confounding and coexposure control; P, precision and internal consistency; and R, reverse causation. L, M, and H denote low, moderate, and high concern, respectively. These domain judgments are qualitative and design-specific; they are not summed into a numerical quality score.

For experimental studies, appraisal considered the Pb compound, route, nominal concentration, duration, achieved blood or tissue dose when reported, species or cell model, metabolic-tissue relevance, directness of the endpoint, use of a metabolic co-stressor, and independent replication. The experimental appraisal separates administered exposure from internal-dose information and explicitly labels cases in which toxicokinetic translation is indeterminate. These domains are descriptive and were not converted into a numerical quality score because the evidence spans incompatible human, animal, and cellular designs.

### 2.3. Explicit Evidence-Interpretation Framework

Mechanistic statements were assigned one of four directness labels. Direct evidence denotes a Pb exposure followed by the stated metabolic event in a relevant cell, tissue, animal, or human study. Supportive evidence denotes a Pb-specific event in another tissue or an adjacent endpoint that strengthens plausibility without demonstrating mediation. Indirect evidence denotes a pathway established in general diabetes or oxidative-stress biology but not directly tested in a Pb-exposed metabolic system. Insufficient evidence denotes an isolated, unreplicated, or materially undercharacterized observation.

Human and experimental evidence were integrated only after this directness classification. Consistency across designs, temporal ordering, dose context, coexposure control, and replication were then used to formulate narrative conclusions. Null findings were interpreted as evidence that an association was not detected under the study’s measurement conditions—not automatically as proof of no effect—while positive findings were examined for residual confounding, reverse causation, multiple testing, and outcome-surrogate limitations.

This procedure improves transparency but remains an author-led critical appraisal, not a formal risk-of-bias instrument, GRADE assessment, systematic review, or meta-analysis. The review therefore avoids pooled causal estimates and uses calibrated terms such as direct, supportive, indirect, preliminary, mixed, and insufficient. Evidence is expressed through pathway-specific descriptions in this synthesis rather than broad confidence grades.

## 3. Epidemiological Evidence: Association Is Inconsistent and Causality Remains Unresolved

The human literature does not show a uniform association between Pb and T2DM-related outcomes. In 3184 Korean adults, blood Pb was not significantly associated with diabetes or HOMA-IR [20]. A retrospective occupational cohort of 34,814 men, including 1035 classified as Pb-exposed, found no clear excess of incident diabetes, although exposure classification and the small number of diabetes cases among exposed workers limited precision [21]. These studies do not support a detectable association under their specific designs, but neither definitively excludes a small, cumulative, nonlinear, or susceptibility-dependent effect.

Positive findings occur more often for surrogate endpoints than for clinical diabetes. In 9645 Korean adults, higher blood Pb was associated with an elevated TyG index, whereas the HOMA-IR analysis was null [22]. Because TyG incorporates triglycerides, this result may reflect lipid perturbation, glucose regulation, or both. Machine-learning feature importance for Pb in a non-diabetic NHANES sample likewise indicates predictive contribution but not causality [23]. A 2025 NHANES analysis found no adjusted positive linear association with HbA1c but reported sex-specific nonlinearity [24]. Collectively, heterogeneity across outcome definitions prevents fasting glucose, HbA1c, TyG, HOMA-IR, metabolic syndrome, and diagnosed T2DM from being treated as interchangeable evidence.

Additional population and review evidence has evaluated Pb in relation to TyG-related indices, metabolic syndrome components, pediatric coexposure settings, obesity or diabetes in environmental-health surveys, and prospective adolescent cardiometabolic outcomes [25,26,27,28,29,30].

The expanded database coverage identified additional population studies that had been omitted from the initial evidence table. Across partly overlapping KNHANES cycles, blood Pb was positively associated with prevalent metabolic syndrome in some analyses, null in others, and often related mainly to triglyceride, blood-pressure, or waist components [31,32,33,34,35]. Because participants and survey years overlap and metabolic syndrome is a composite surrogate, these reports should not be counted as five independent demonstrations of Pb-related diabetes.

Other studies likewise show endpoint and exposure dependence. Urinary Pb was positively associated with glucose, insulin, HOMA-IR, and metabolic syndrome in Taiwanese youth [36]. Canadian data showed only small fasting-glucose differences and no higher odds of dysglycemia by fasting-glucose or HbA1c thresholds [37], whereas a southern-Taiwan study reported a small positive HbA1c association [38]. Repeated blood measurements in 5505 Chinese adults showed higher fasting glucose and lower HOMA-β over five years in women but not men [39]. This prospective sex-specific study strengthens temporality but does not establish clinical T2DM.

Life-course and contextual studies add further heterogeneity. Repeated early-life Pb biomarkers in 601 Mexican children were associated mainly with lipid, anthropometric, and blood-pressure indicators rather than consistent glycemia [40]. Urinary Pb in 734 Chinese adults was associated cross-sectionally with impaired fasting glucose and T2DM in a multi-metal network analysis [41]. A mining-area study found no Pb–fasting-glucose association [42], whereas an NHANES analysis reported a depression-dependent urinary Pb–T2DM interaction [43]. A Pakistani Pb/nickel study reported metabolic associations but could not isolate Pb cleanly [44]. These findings enlarge the evidence base without resolving causality.

Pediatric and life-course evidence is also context-dependent. In children near an e-waste recycling area, Pb and PM2.5 changed alongside lockdown-related behavior and inflammatory markers, preventing Pb-specific attribution [28]. In a prospective Mexico City birth cohort, prenatal blood Pb was not positively associated with glucose, insulin, or HOMA-IR at 10–18 years [30]; however, prenatal Pb is not a measure of the child’s cumulative or concurrent adolescent burden. These results are informative for the exposure windows studied, but they do not resolve effects of adult or lifelong exposure.

The direction of bias can differ across studies. Residual confounding by smoking, occupation, housing, diet, renal function, iron status, socioeconomic conditions, or other toxicants may inflate a positive association. Conversely, a single blood-Pb measurement, within-person variability, an occupational job-category proxy, outcome misclassification, and restricted exposure contrast can attenuate an association toward the null. Cross-sectional disease-related changes in behavior, bone turnover, renal handling, or erythrocyte kinetics can also generate reverse causation in either direction. Table 1 therefore evaluates positive and null studies with the same causal criteria.

Figure 2 summarizes the direction and principal limitations of the human and experimental evidence considered in this review.

### 3.1. Exposure Measurement, Contemporary Exposure Range, and Bias Direction

Blood Pb reflects recent exposure plus release from bone, whereas T2DM develops over years. A single blood measurement can therefore misclassify long-term etiologically relevant exposure because of within-person variability, changing sources, and endogenous mobilization. Nondifferential misclassification generally attenuates associations; this is particularly important for null studies that used one blood sample or a broad occupational category. Repeated blood measures and bone Pb would better separate recent from cumulative burden, but they remain uncommon in the metabolic literature.

Contemporary population studies frequently operate within low and compressed blood-Pb distributions, often in the low-single-digit microgram-per-deciliter range [24,25,26]. Reduced contrast can weaken statistical signals even if no safe threshold is assumed, especially for nonlinear effects or susceptible subgroups. This possibility should not be used to dismiss null estimates; rather, it defines what those estimates can exclude. Equally, low-level positive associations demand careful control for socioeconomic patterning, smoking, renal function, diet, other metals, and model flexibility. The appropriate interpretation is symmetrical: null results may be attenuated, while positive results may be inflated or noncausal.

### 3.2. Lead Within Real-World Exposure Mixtures

Lead rarely occurs as an isolated exposure. Shared sources and social conditions create correlation with cadmium, arsenic, mercury, manganese, PM2.5, poor nutrition, occupational hazards, psychosocial stress, and material deprivation [4,5,6,23,26,28]. These factors can act as confounders, effect modifiers, or joint causal components. A conventional single-pollutant coefficient may therefore represent a lead-specific contribution, residual mixture confounding, or the effect of an exposure pattern that cannot be decomposed with the available data.

The studies summarized here vary substantially in coexposure control. Multi-metal surveys often model individual metals despite correlated biomarker profiles, and the e-waste/lockdown study jointly implicates Pb, particulate pollution, and contextual change [23,28]. Future cohorts should prespecify a causal structure, measure major co-pollutants repeatedly, report both single- and multi-pollutant estimates, test biologically justified interactions, and use mixture-aware models with adequate sample size. Until such data are available, the most defensible conclusion is that Pb may be one component of a diabetogenic exposure environment; its independent contribution remains unresolved.

### 3.3. Geographic Context and the Mexican Evidence Gap

Mexico is retained as a brief illustrative context rather than a special evidentiary focus. Mining, smelting, contaminated dust, informal recycling, occupational activity, and lead-glazed ceramics make exposure prevention important [15], yet the search identified only one prospective Mexican study with diabetes-related outcomes: prenatal Pb was not positively associated with adolescent glucose, insulin, or HOMA-IR [30]. Mexican studies of hepatic steatosis and oxidative stress provide contextual but not diabetes-specific evidence [45,46]. This mismatch between high exposure relevance and sparse longitudinal metabolic data motivates locally grounded, mixture-aware cohorts; it does not justify a country-specific causal conclusion.

## 4. Experimental Evidence and Dose Interpretation

### 4.1. Acute Lethality and Chronic Metabolic Relevance

There is no single LD_50_ for elemental Pb or ‘lead’ as a class. Acute lethality depends on chemical species, hydration state, route, vehicle, sex, species, and observation period. For lead(II) acetate trihydrate, a commonly cited oral-rat LD_50_ is 4665 mg/kg [47,48], whereas inhalation is governed by airborne concentration and particle characteristics rather than an oral mass-per-body-weight value [49]. These values identify acute hazard and should not be used as a benchmark for chronic metabolic effects.

The relevant translational question is not whether a repeated exposure is below an LD_50_, but whether its absorbed and tissue dose resembles the internal exposure of the population being discussed. Administered drinking-water concentration, percentage solution, oral dose, cell-culture molarity, whole-blood Pb, and tissue Pb are different metrics. They cannot be converted reliably without compound identity, intake, absorption, distribution, age, bone storage, and clearance data. The dose appraisal therefore records the achieved internal dose when available and otherwise labels translation as indeterminate.

### 4.2. Dose Context and Organ-Specific Findings

The experimental evidence considered here spans direct β-cell studies, adjacent β-cell and endoplasmic-reticulum biology, and animal models of hepatic or gut–metabolic effects [50,51,52,53,54,55,56,57,58,59]. The contrast between experimental and human exposure metrics is substantial. Recent population studies generally evaluate whole-blood Pb in low-single-digit micrograms per deciliter, whereas representative experiments use 10–25 micromolar Pb in cell medium, 0.05–0.2% Pb-containing drinking-water solutions, or 200 mg/L Pb for months [24,25,26,50,51,57,58,59]. These nominal concentrations are not directly comparable to whole-blood Pb because protein binding, erythrocyte partitioning, route, and species toxicokinetics differ. Where achieved blood or tissue Pb was not reported, environmental relevance remains indeterminate rather than assumed.

Direct experimental support is clearest for β-cell bioenergetics and hepatic glucose production. Isolated mouse β-cells exposed to Pb showed reduced ATP production, mitochondrial respiration, membrane potential, and glucose-stimulated insulin secretion; the reported ATP EC50 was 25 micromolar, with effects on some measures at 10 micromolar [50]. Rat islets and Pb-treated rats showed impaired insulin secretion, glucose intolerance, and altered hepatic gluconeogenesis after a 32-day design that included 0.2% Pb exposure [51]. Additional β-cell models support impaired stimulated insulin secretion and functional β-cell mass [52]. These observations demonstrate hazard under the tested conditions but do not by themselves establish effects at contemporary environmental exposure.

Hepatic and gut–metabolic studies also require dose and co-stressor qualification. Rats given 0.05% Pb in drinking water for 28 weeks developed higher fasting glucose and hepatic glucose production with increased G6PC, FBP1, and PCK1 expression [57]. In high-fat-diet mice, 200 mg/L Pb for 24 weeks worsened glucose and lipid phenotypes and produced an achieved blood Pb concentration of approximately 10 micrograms per deciliter [58]. A related high-fat-diet model reported gut-barrier disruption, microbiota changes, and transfer of metabolic impairment through fecal microbiota transplantation [59]. These findings support interaction with dietary susceptibility; the limited effect in normal-diet animals and related research groups constrain generalization and independent replication.

A separate rat study reported weight gain and DNA hypermethylation after Pb exposure [60]. Because food intake, body composition, energy expenditure, and systemic glucose outcomes were not jointly characterized, weight gain alone was not classified as metabolic toxicity or evidence of diabetes. Broader reviews of metal-related mitochondrial toxicity and metabolomic disruption provide mechanistic context but do not constitute direct evidence of Pb-induced dysglycemia [61,62].

Pb-induced changes in hippocampal insulin signaling and glucose metabolism provide neural evidence but are not equivalent to systemic T2DM [63]. Across models, recurrent limitations include high administered concentrations, incomplete internal-dose reporting, route-dependent toxicokinetics, small or sex-restricted groups, and use of a high-fat diet. Consequently, experimental studies establish biological plausibility and identify candidate organs, but human relevance should be anchored to measured blood or tissue Pb, physiologically based dose modeling, and responses across environmentally plausible internal-dose ranges. Table 2 summarizes these dose-context and translational limitations across representative experiments.

Figure 3 depicts the proposed organ-level pathways and distinguishes directly demonstrated or established relationships from indirect or incompletely demonstrated links.

## 5. Redox Mechanisms: Nrf2/Keap1 and the Thioredoxin System

### 5.1. Pb-Induced Oxidative Stress

Pb can induce oxidative stress indirectly through sulfhydryl binding, displacement of essential-metal cofactors, impaired antioxidant enzymes and glutathione homeostasis, mitochondrial dysfunction, and delta-aminolevulinate accumulation [64,65,66,67,68,69]. These effects are well supported as general Pb toxicology, but oxidative stress is neither specific to diabetes nor sufficient to establish a Pb-to-T2DM pathway.

Human and animal studies report altered catalase, glutathione-related markers, and oxidative damage after Pb exposure [70,71,72,73]. Their metabolic interpretation is limited because many biomarkers are nonspecific and have rarely been linked prospectively to a clinical diabetes endpoint. Oxidative stress is therefore treated here as a supportive molecular initiating event, not as direct evidence of diabetogenicity. Genotoxic, AhR-related, and epigenetic responses have also been discussed in broader Pb and heavy-metal toxicology [74,75,76], but their relevance to glucose regulation remains indirect.

### 5.2. The Nrf2/Keap1 Antioxidant Axis

Nrf2 is regulated by Keap1-dependent turnover and can induce antioxidant and detoxification genes under oxidative or electrophilic stress [77,78,79]. Pb-related Nrf2 responses vary by dose, duration, tissue, and compensatory state: transient activation may be protective, whereas sustained injury may coincide with inadequate nuclear Nrf2 signaling.

Pb-specific metabolic mediation through Nrf2 has not been established. In PC12 cells, 10 micromolar Pb for 24 h increased ROS, malondialdehyde, Keap1, and TXNIP while decreasing Nrf2 and antioxidant measures; peptide pretreatment partly reversed these changes [80]. This directly demonstrates a Pb-responsive redox pattern in a neural cell line, not an Nrf2-mediated glucose phenotype in β-cells, liver, muscle, adipose tissue, or humans.

General β-cell biology makes Nrf2 mechanistically relevant because β-cells have limited antioxidant reserve and tightly regulated ROS signaling [81,82,83,84]. However, moving from that background to a claim that Nrf2 mediates Pb-associated glucose dysregulation requires Pb-exposed metabolic-tissue experiments with pathway perturbation, rescue, and dose-matched controls. Those data are currently sparse.

Accordingly, Nrf2 is classified as indirect for the Pb-to-glucose link: Pb-specific redox effects are supportive, but metabolic mediation remains unproven. This distinction prevents general redox plausibility from being read as direct Pb-metabolic evidence.

### 5.3. Thioredoxin, Thioredoxin Reductase, and TXNIP

The thioredoxin system—thioredoxin, thioredoxin reductase, and peroxiredoxins—maintains protein-thiol redox balance. Pb can bind sulfhydryl- and selenol-containing proteins, and renal studies show changes in thioredoxin-reductase activity after Pb exposure [85,86]. These are direct Pb-redox observations, but the kidney findings do not establish altered glucose regulation.

TXNIP can inhibit thioredoxin and, in general stress biology, participate in NLRP3 inflammasome activation, interleukin-1β maturation, β-cell injury, and insulin resistance [87,88,89,90,91,92,93]. The Pb-exposed PC12-cell study reported increased TXNIP [80], but the sequence Pb to TXNIP to NLRP3 to β-cell dysfunction has not been directly demonstrated in a Pb-exposed metabolic tissue.

The thioredoxin/TXNIP/NLRP3 module is therefore classified as indirect or insufficient for Pb-associated diabetes. It is a testable hypothesis rather than an established mediator. Priority experiments should combine environmentally anchored internal doses with metabolic-tissue-specific TXNIP or NLRP3 perturbation and rescue; without such tests, general oxidative-stress biology should not be presented as Pb-specific proof.

## 6. Evidence Directness Across Mechanistic Modules

Mechanistic coherence is not equivalent to evidentiary independence. This synthesis therefore asks a narrower question for each module: what event was directly observed after Pb exposure, in which biological context, and what untested step separates that observation from human glucose dysregulation? This approach prevents general redox or diabetes pathways from being counted as if they were Pb-specific metabolic demonstrations.

### Pathway-Specific Evidence Status

Direct experimental evidence supports β-cell mitochondrial impairment and reduced stimulated insulin secretion [50,51,52], as well as increased hepatic gluconeogenesis in a limited number of rodent and cell models [51,57]. The corresponding human evidence remains mixed, and most experimental designs lack a toxicokinetic bridge to contemporary population exposure. These modules are therefore described as directly demonstrated under experimental conditions but translationally limited—not assigned an unqualified confidence grade.

By contrast, Pb-specific metabolic evidence for Nrf2 mediation is indirect; TXNIP/NLRP3-mediated β-cell dysfunction is untested as a complete Pb-driven sequence; and gut-microbiota mediation is preliminary and largely derived from related high-fat-diet mouse studies [58,59,80,87,88]. These pathways remain biologically plausible hypotheses. Independent replication, pathway perturbation, tissue-specific measurements, and internal-dose anchoring are required before they can be described as established Pb-metabolic mechanisms. Table 3 summarizes the directness and principal limitations of each proposed mechanism.

## 7. Other Endocrine and Lipid Pathways: Supportive but Not Specific

Pb exposure has been associated with hypothalamic–pituitary–adrenal (HPA) perturbation, but direction and magnitude vary by age, sex, timing, occupational context, stress protocol, and biomarker. A systematic review concluded that findings across the life course are heterogeneous [94]. In a small middle-aged sample, blood Pb was associated with neither diurnal nor stress-reactive salivary cortisol, whereas occupational and older-adult studies reported altered stress-hormone profiles or allostatic load [95,96,97]. HPA effects should therefore be treated as context-dependent modifiers, not a confirmed pathway from Pb to diabetes.

Thyroid-axis findings are also heterogeneous. An updated meta-analysis, studies of lead-poisoned patients and exposed communities, and an in vivo/in silico analysis report variable changes across thyroid measures [98,99,100,101]. Thyroid-stimulating hormone (TSH) is a glycoprotein secreted by the anterior pituitary that stimulates the thyroid gland; it is not a thyroid hormone. Current evidence does not justify a deterministic sequence of Pb increased glucagon, reduced basal metabolism, and diabetes. Human observations of leptin and other endocrine mediators likewise do not establish metabolic mediation [102].

Lipid metabolism may be more consistently associated with blood Pb than clinical diabetes itself. NHANES and occupational studies report hyperlipidemia or altered lipid profiles, and Pb can promote adipocyte differentiation in vitro [103,104,105,106]. These findings may partly explain associations with TyG, which incorporates triglycerides, but should be described as metabolic dysfunction rather than dysglycemia unless glucose regulation is also measured. Endocrine and lipid observations are therefore supportive context and potential effect-modifying pathways, not independent proof of Pb diabetogenicity.

## 8. Causal Interpretation, Research Priorities, and Conclusions

Across evidence streams, biological plausibility is stronger than epidemiological causal inference. Pb directly impairs β-cell bioenergetics and insulin secretion and can increase hepatic gluconeogenesis under experimental conditions [50,51,52,57]. However, the human literature is inconsistent and often relies on a single blood-Pb measurement, cross-sectional timing, heterogeneous surrogate endpoints, and incomplete coexposure control. Null estimates may be attenuated by exposure misclassification or restricted low-level contrast; positive estimates may reflect residual confounding, reverse causation, lipid-sensitive surrogates, or model flexibility. Both require symmetrical interpretation.

Mechanistic confidence must also be pathway-specific. General Pb-induced oxidative stress is well supported but is not diabetes-specific. Nrf2-mediated metabolic effects remain indirect, the proposed Pb–TXNIP–NLRP3–β-cell sequence is unproven, and gut-microbiota mediation is preliminary and largely confined to related HFD mouse models. Drinking-water percentages and cell-medium molarity cannot substitute for measured internal dose. Future experiments should report blood and tissue Pb, test multiple environmentally anchored internal doses, include both sexes and relevant susceptibility states, and demonstrate necessity or rescue of proposed mediators.

The most informative human studies would be prospective and mixture-aware, with repeated blood Pb plus a cumulative marker such as bone Pb; adjudicated incident T2DM; repeated fasting glucose, insulin, HbA1c, and lipid measures; and prespecified control of kidney function, iron status, smoking, diet, socioeconomic conditions, other metals, particulate pollution, and psychosocial stress. Current evidence supports Pb as a plausible component of a broader diabetogenic exposure environment under some exposure and susceptibility conditions, but does not establish environmental Pb as an independent cause of human T2DM.

## Figures and Tables

**Figure 1:**
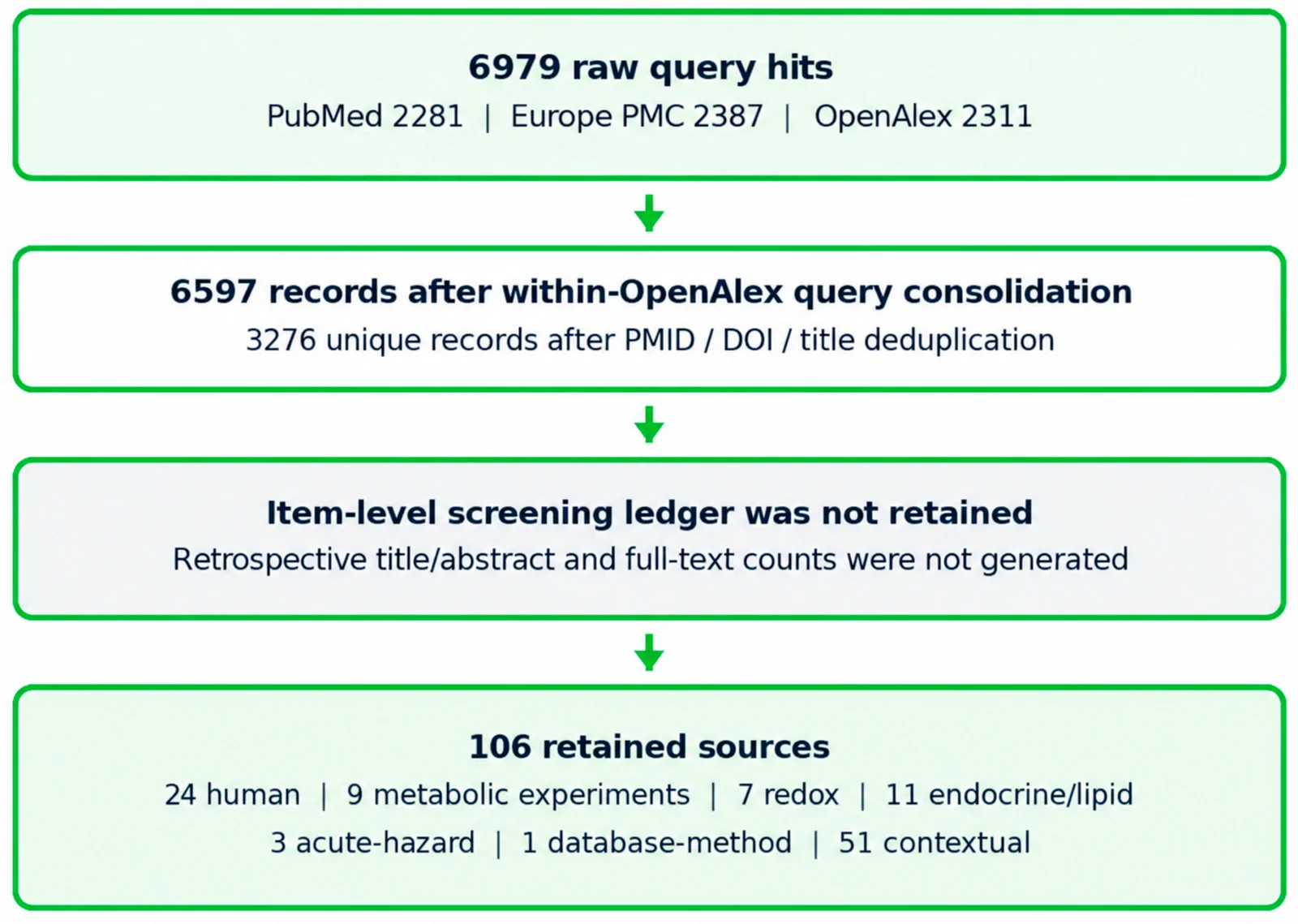
Transparent source-accounting flow for this author-led critical review. Exact retrieval and deduplication counts are reported. Because the original review did not retain a contemporaneous item-level screening ledger, title/abstract, full-text, and reason-specific exclusion counts were not retrospectively reconstructed.

**Figure 2:**
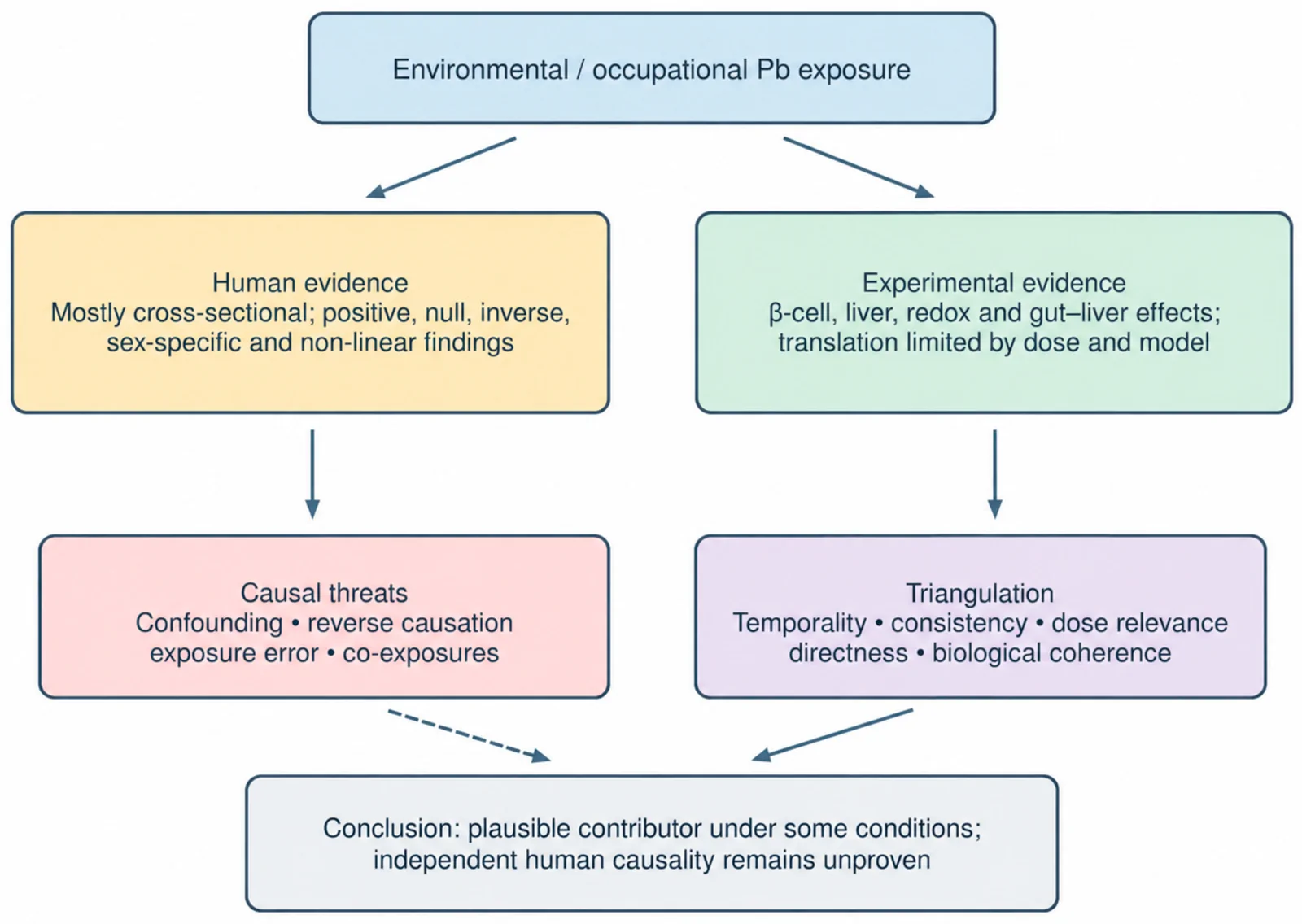
Critical evidence synthesis for Pb and glucose dysregulation. Human findings are mixed and predominantly cross-sectional; both positive and null estimates can be affected by exposure timing, outcome definition, residual confounding, coexposure, and restricted contemporary exposure contrast. Experimental studies provide biological plausibility but require dose and model qualification.

**Figure 3:**
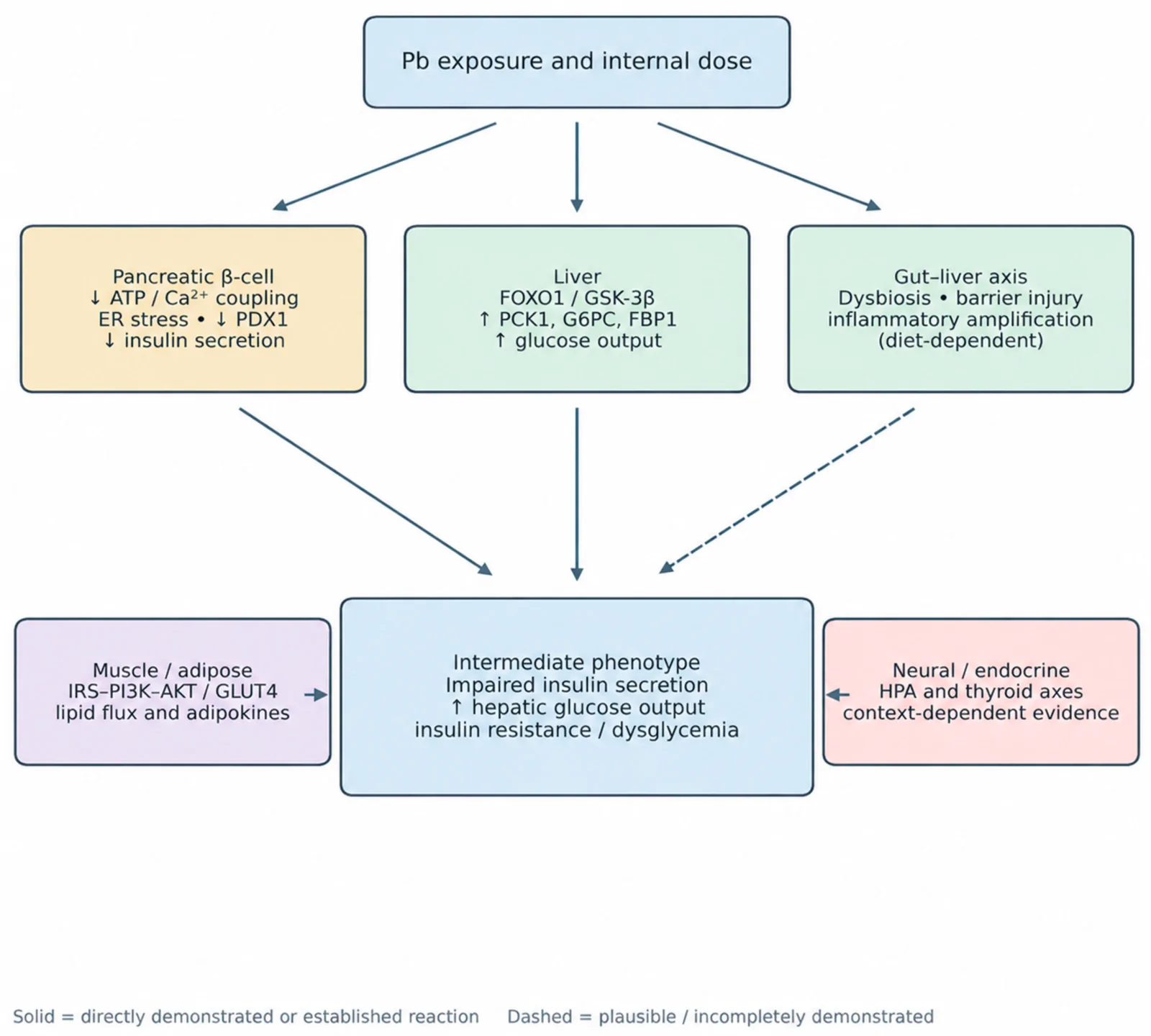
Organ-level pathways proposed for Pb-associated glucose dysregulation. Solid relationships represent events demonstrated in Pb-exposed metabolic models; dashed relationships are cross-organ or general-diabetes links that remain indirect for Pb. The figure illustrates biological plausibility, not relative evidence strength or human dose relevance. Arrowheads indicate the proposed direction of biological influence.

**Table 1:** Key human studies and direction-aware appraisal of Pb-related metabolic findings.

Study/Design	Population	Pb Assessment	Outcome and Principal Result	Balanced Appraisal and Likely Bias Direction
Moon 2013 [20] Cross-sectional KNHANES	3184 adults ≥ 30 y, Korea	Whole-blood Pb	No significant association with diabetes or HOMA-IR	A single recent-exposure measure and cross-sectional design can attenuate a cumulative effect; residual multi-metal confounding can bias either direction. Appraisal profile (T/E/O/C/P/R): H/H/M/M/M/H.
Ji et al. 2021 [21] Retrospective occupational cohort	34,814 men; 1035 Pb-exposed, Korea	Occupational category and blood Pb	Incident DM: HR 1.01 (0.58–1.77); very small correlation with annual fasting-glucose change	Few DM cases and job-category misclassification reduce precision and can dilute an association; male workforce limits generalizability. Appraisal profile (T/E/O/C/P/R): M/H/L/M/H/L.
Moon et al. 2022 [29] Cross-sectional KoNEHS	3787 adults, Korea	Blood Pb	Pb associated with obesity, but not increased DM; inverse DM association in men	Medication-defined DM, selection, and disease-related toxicokinetic or behavioral change permit outcome misclassification and reverse causation. Appraisal profile (T/E/O/C/P/R): H/H/M/M/M/H.
Jin et al. 2024 [22] Cross-sectional KNHANES	9645 adults; HOMA-IR subset n = 3678	Whole-blood Pb	High TyG OR 1.32–1.46 for Q4 vs. Q1; stronger in men; HOMA-IR null	TyG partly reflects lipids. A single Pb measure may attenuate cumulative effects, whereas residual confounding can inflate a positive association. Appraisal profile (T/E/O/C/P/R): H/H/M/M/L/H.
Liu et al. 2024 [23] Cross-sectional NHANES/ML	4354 non-diabetic adults, USA	Blood heavy metals	Pb contributed to an interpretable IR prediction model	Feature importance is not a causal effect; cross-sectional selection, data-driven thresholds, and correlated metals complicate attribution. Appraisal profile (T/E/O/C/P/R): H/H/H/H/M/H.
Wang et al. 2025 [24] Cross-sectional NHANES	12,049 adults, USA	Whole-blood Pb	No adjusted positive linear HbA1c association; sex-specific nonlinearity reported	Low exposure contrast and a single Pb measure can obscure small or nonlinear effects; HbA1c is affected by erythrocyte biology and the design is cross-sectional. Appraisal profile (T/E/O/C/P/R): H/H/M/M/L/H.
Park et al. 2019 [26] Cross-sectional KNHANES	2833 adults, Korea	Blood Pb	Pb correlated with fasting glucose, triglycerides, and other metabolic-syndrome components	Component-wise testing and confounding may inflate signals; a single BLL may attenuate chronic effects; metabolic syndrome is not incident T2DM. Appraisal profile (T/E/O/C/P/R): H/H/H/M/M/H.
Zheng et al. 2024 [28] Before/during-lockdown comparison	235 children aged 3–7 y, China	Blood Pb plus PM2.5	Lower exposures during lockdown coincided with lower fasting glucose and HOMA-IR; inflammation pattern was complex	Pb, PM2.5, behavior, and context changed together; neither a Pb-specific effect nor absence of one can be isolated. Appraisal profile (T/E/O/C/P/R): H/M/M/H/M/H.
Liu et al. 2020 [30] Prospective birth cohort	369 offspring aged 10–18 y, Mexico City	Repeated maternal blood Pb during pregnancy	No positive glucose/insulin/HOMA-IR signal; lower lipids in boys at ≥5 μg/dL	Prospective timing helps, but prenatal BLL is not adolescent or cumulative burden; attrition and modest size may obscure small sex-specific effects. Appraisal profile (T/E/O/C/P/R): L/M/M/M/M/L.
Hu et al. 2025 [25] Cross-sectional NHANES	US adults in a multi-metal analysis	Blood heavy metals	Pb was examined in relation to TyG-related indices and inflammatory mediation	Cross-sectional mediation cannot establish temporal order; correlated metals and surrogate outcomes complicate attribution. Appraisal profile: H/H/M/H/M/H.
Rhee et al. 2013 [31] Cross-sectional KNHANES 2008	1405 adults ≥ 20 y, Korea	Whole-blood Pb and other metals	Highest Pb quartile associated with prevalent metabolic syndrome (OR 2.57, 95% CI 1.46–4.51)	Composite outcome, cross-sectional temporality, and multi-metal setting; overlapping national survey evidence. Appraisal profile: H/H/H/M/M/H.
Lee and Kim 2013 [32] Cross-sectional KNHANES 2005–2010	Nationally representative Korean adults	Blood Pb, Cd, and Hg	Adjusted Pb association with metabolic syndrome was not robust	Single biomarker and composite endpoint; overlapping survey cycles limit independence. Appraisal profile: H/H/H/M/M/H.
Moon 2014 [33] Cross-sectional KNHANES 2009–2010	3950 adults, Korea	Blood Pb with other metals	Modest Pb–metabolic-syndrome association; combined-metal score showed stronger association	Mixture result limits Pb-specific attribution; overlapping survey participants. Appraisal profile: H/H/H/H/M/H.
Choi et al. 2020 [34] Cross-sectional KNHANES 2013	1688 adults ≥ 19 y, Korea	Blood Pb	Pb associated with metabolic syndrome; TSH did not explain the association	Cross-sectional mediation and composite outcome; modest sample and residual confounding. Appraisal profile: H/H/H/M/M/H.
Park and Oh 2021 [35] Cross-sectional KNHANES 2016	823 adults aged 19–29 y, Korea	Blood Pb, Hg, and Cd	Pb related to selected blood-pressure, waist, and triglyceride components	Young sample and composite components do not establish diabetes; limited precision. Appraisal profile: H/H/H/M/H/H.
Lin et al. 2020 [36] Cross-sectional	738 adolescents and young adults, Taiwan	Urinary Pb	Higher urinary Pb associated with glucose, insulin, HOMA-IR, and metabolic syndrome	Spot urine, cross-sectional timing, and correlated exposures limit causal interpretation. Appraisal profile: H/H/M/M/M/H.
Ngueta and Kengne 2017 [37] Cross-sectional CHMS	Canadian adults	Whole-blood Pb	Small fasting-glucose difference; no higher dysglycemia odds by fasting glucose or HbA1c	Low exposure contrast and one blood measure may attenuate small effects; outcome definitions were explicit. Appraisal profile: H/H/M/M/M/H.
Chang et al. 2021 [38] Cross-sectional	Non-diabetic adults, southern Taiwan	Blood Pb	A log 1 μg/dL increase in Pb associated with a small HbA1c increase	HbA1c can be influenced by erythrocyte kinetics; cross-sectional design. Appraisal profile: H/H/M/M/M/H.
Wang et al. 2022 [39] Repeated-measures cohort	5505 Chinese adults followed 5 y	Repeated blood Pb	Women had higher fasting glucose and lower HOMA-β; men were null	Repeated exposure and follow-up improve temporality, but sex-specific surrogate changes require replication. Appraisal profile: M/M/M/M/L/M.
Muciño-Sandoval et al. 2021 [40] Prospective birth cohort	601 mother–child dyads, Mexico	Repeated prenatal/early-childhood blood Pb and maternal bone Pb	Mainly lipid, anthropometric, and blood-pressure signals; glycemic findings were not consistent	Prospective repeated biomarkers are a strength; multiple windows/endpoints and attrition remain. Appraisal profile: L/M/H/M/M/L.
Wang et al. 2023 [41] Cross-sectional multi-metal study	734 adults, China	Urinary metals including Pb	Pb associated with impaired fasting glucose and T2DM in a data-driven network	Spot urine, cross-sectional disease status, and metal correlation prevent Pb-specific causal attribution. Appraisal profile: H/H/M/H/M/H.
Luthviatin et al. 2024 [42] Cross-sectional mining-area study	Women aged 30–49 y, Indonesia	Blood Pb	No association between blood Pb and fasting blood sugar	Purposive, small mining-area sample and one outcome yield limited precision/generalizability. Appraisal profile: H/H/M/H/H/H.
Wang et al. 2024 [43] Cross-sectional NHANES 2007–2018	US adults stratified by depression	Urinary Pb, Cd, and Hg	Urinary Pb–T2DM association observed mainly among participants with depression	Interaction is data-dependent; spot urine, multiple testing, and cross-sectional timing limit inference. Appraisal profile: H/H/L/H/M/H.
Feezan et al. 2025 [44] Cross-sectional	2688 participants, Pakistan	Urinary and groundwater Pb and Ni	Pb-detected diabetic participants had higher Pb and glycemic biomarkers	Case-grouping, cross-sectional timing, and Pb/Ni coexposure do not isolate a Pb-specific effect. Appraisal profile: H/H/L/H/M/H.

Abbreviations: CI, confidence interval; DM, diabetes mellitus; HbA1c, glycated hemoglobin; HOMA-IR, homeostatic model assessment of insulin resistance; HR, hazard ratio; IR, insulin resistance; ML, machine learning; OR, odds ratio; Q, quartile; TyG, triglyceride-glucose index. ‘Balanced appraisal’ identifies limitations that could attenuate, inflate, or otherwise complicate the reported association; it is not a numerical risk-of-bias score. Domain key: T, temporality; E, exposure; O, outcome; C, confounding/coexposure; P, precision/consistency; R, reverse causation; L/M/H, low/moderate/high concern.

**Table 2:** Administered exposure, internal-dose information, and translational interpretation in representative Pb-metabolic experiments.

Study/Model	Administered Exposure	Internal-Dose Information	Key Metabolic Result	Translational Interpretation
Mostafalou et al. [51] Rat islets + rats	Islets exposed for 24 h; rats received lead acetate for 32 d, including 0.2% Pb	BLL increased in the 0.2% group; exact achieved value not reproduced here	Reduced insulin secretion; glucose intolerance; increased gluconeogenesis	Direct pancreatic/hepatic hazard evidence, but high administered concentration and short duration limit contemporary relevance.
Elmorsy et al. [50] CD-1 mouse β-cells	Pb in vitro; ATP EC50 25 μM; some effects at 10 μM	Not applicable: nominal cell-medium concentration	Reduced glucose-stimulated insulin secretion and mitochondrial function	Cell-medium molarity cannot be equated with total whole-blood Pb; isolated-cell exposure likely overstates freely available circulating Pb.
Daniel et al. [52] β-cell models	Pb2+ in vitro and in vivo	Achieved blood/tissue dose not reported in the present synthesis	Reduced stimulated insulin secretion and functional β-cell mass	Direct β-cell evidence; environmental translation remains indeterminate without matched internal dose.
Wan et al. [57] Wistar rats	0.05% Pb in drinking water for 28 wk	Achieved BLL not reported in the present synthesis	Higher fasting glucose and hepatic glucose production; G6PC, FBP1, and PCK1 increased	A 0.05% solution is a high nominal water concentration; absorption and blood/tissue dose are required for human comparison.
Wang et al. [58] HFD mice	200 mg/L Pb in drinking water for 24 wk, with or without HFD	Approximately 10 μg/dL blood Pb reported	Pb worsened fasting glucose, insulin resistance, and hepatic glucolipid injury mainly with HFD	Internal BLL overlaps some historically exposed populations, but administered dose, species kinetics, and strong diet interaction limit generalization.
Wang et al. [59] HFD mice	200 mg/L Pb-containing water in a related HFD model	Approximately 10 μg/dL BLL reported for this model system	Gut dysbiosis, barrier damage, and transferred glucose impairment	Preliminary mediation evidence; HFD dependence, facility-sensitive microbiota, and related investigators limit independent replication.
Yun et al. [63] Juvenile rats	0.1–0.3% lead acetate orally for 65 d	Achieved blood/tissue Pb not reported in the present synthesis	Altered hippocampal glucose metabolism and insulin signaling	High nominal exposure and a neural endpoint do not establish systemic T2DM at environmental dose.
Sun et al. [60] Adult rats	Lead acetate exposure; repeated-dose rat model	Achieved blood/tissue dose not reproduced in the present synthesis	Weight gain accompanied by DNA hypermethylation	Weight gain alone was not classified as metabolic toxicity because food intake, body composition, energy expenditure, and systemic glucose outcomes were not jointly characterized.

Nominal medium concentration, drinking-water concentration, and whole-blood Pb are different exposure metrics and should not be compared as if equivalent. ‘Not reported here’ means that the cited article’s achieved internal dose was unavailable for the present synthesis; it does not imply that no toxicokinetic data exist elsewhere. BLL, blood lead level; EC50, half-maximal effective concentration; ER, endoplasmic reticulum; HFD, high-fat diet.

**Table 3:** Directness and limitations of proposed Pb-associated glucose-regulatory mechanisms.

Mechanistic Module	Direct Pb-Specific Observation	Evidence Status for Pb to Glucose	Principal Limitation or Missing Test
Molecular initiation/oxidative stress	Thiol binding, metal displacement, mitochondrial disturbance, and redox-marker changes after Pb exposure [11,50,64,65,66,67,68,69,70,71,72,73]	Direct for general Pb toxicity; supportive but nonspecific for glucose dysregulation	Oxidative stress is common to many toxicants and diseases; prospective metabolic mediation is rarely tested.
β-cell bioenergetics and secretion	Reduced ATP, respiration, membrane potential, stimulated insulin secretion, and functional β-cell mass in Pb models [50,51,52]	Direct experimental evidence; translational relevance limited	Predominantly isolated cells or rodents; nominal concentrations are high and matched human internal-dose evidence is lacking.
Hepatic gluconeogenesis	Higher hepatic glucose production and increased PCK1/G6PC/FBP1 in Pb-treated models [51,57]	Direct experimental evidence from few models	Limited independent replication; administered dose and achieved internal dose are incompletely linked to contemporary exposure.
Nrf2/Keap1 metabolic mediation	Nrf2/Keap1 changes reported in Pb-exposed PC12 and other non-metabolic toxicity models [77,78,79,80]	Indirect for glucose regulation	No clear demonstration that Nrf2 perturbation mediates a Pb-induced phenotype in β-cell, liver, muscle, adipose tissue, or humans.
Thioredoxin/TXNIP/NLRP3	Pb alters renal TrxR and increases TXNIP in PC12 cells [80,85,86]; TXNIP–NLRP3 biology is established generally [87,88]	Indirect to insufficient for Pb-associated β-cell dysfunction	The complete Pb to TXNIP to NLRP3 to metabolic-tissue injury sequence has not been directly tested.
Gut–liver axis	Dysbiosis, barrier injury, and fecal-transfer effects in Pb-exposed HFD mice [58,59]	Preliminary and model-dependent	HFD dependence, facility-sensitive microbiota, related investigators, and limited independent replication.
Human dysglycemia/T2DM	Mixed associations across incident DM, fasting glucose, HbA1c, TyG, and HOMA-IR [20,21,22,23,24,25,26,27,28,29,30]	Limited and inconsistent	Mostly cross-sectional; heterogeneous outcomes, single BLLs, coexposures, and residual confounding prevent causal attribution.

Evidence status describes directness for the specific Pb-to-glucose proposition, not general biological plausibility and not a formal GRADE score. Direct experimental evidence can still have low human relevance when dose, model, or replication is limited. Every module is linked to the Pb-specific and bridge references used for its classification.

## Data Availability

The original contributions presented in this study are included in the article. Further inquiries can be directed to the corresponding author.

## Abbreviations

**Abbreviation**	**Full name**
ARE	antioxidant response element
BLL	blood lead level
CI	confidence interval
DM	diabetes mellitus
EC_50_	half-maximal effective concentration
GSH	reduced glutathione
HbA1c	glycated hemoglobin
HFD	high-fat diet
HOMA-IR	homeostatic model assessment of insulin resistance
HPA	hypothalamic–pituitary–adrenal
IR	insulin resistance
Keap1	Kelch-like ECH-associated protein 1
LD_50_	median lethal dose
ML	machine learning
NLRP3	NOD-like receptor family pyrin domain-containing 3
Nrf2	nuclear factor erythroid 2-related factor 2
OR	odds ratio
Pb	lead
ROS	reactive oxygen species
T2DM	type 2 diabetes mellitus
Trx	thioredoxin
TrxR	thioredoxin reductase
TSH	thyroid-stimulating hormone
TXNIP	thioredoxin-interacting protein
TyG	triglyceride-glucose index

## Appendix A. Database-Specific Search Strategies

Searches were executed on 4 August 2026 from database inception through that date; the supplementary OpenAlex search was executed on 6 September 2026 with the same publication cutoff. Europe PMC synonym expansion was disabled. Counts are raw query yields and overlap across databases and search families; they are not unique included-study counts. Retrieval, within-OpenAlex consolidation, and cross-source deduplication are described in Section 2.1 and Figure 1.

### Appendix A.1. Metabolic/Epidemiological

PubMed/MEDLINE (483 records): (“lead exposure”[tiab] OR “blood lead”[tiab] OR “bone lead”[tiab] OR “urinary lead”[tiab] OR “lead acetate”[tiab] OR “lead poisoning”[tiab] OR “lead toxicity”[tiab] OR lead-induced[tiab] OR “Pb exposure”[tiab] OR Pb-induced[tiab] OR “Pb toxicity”[tiab] OR plumbism[tiab]) AND (“Diabetes Mellitus, Type 2”[Mesh] OR diabet*[tiab] OR glucose[tiab] OR “insulin resistance”[tiab] OR HOMA-IR[tiab] OR “triglyceride-glucose”[tiab] OR TyG[tiab] OR “metabolic syndrome”[tiab] OR “pancreatic beta cell”[tiab] OR “pancreatic β-cell”[tiab] OR gluconeogenesis[tiab]) AND (“1900/01/01”[dp]: “2026/08/04”[dp]).

Europe PMC (498 records): (TITLE_ABS:”lead exposure” OR TITLE_ABS:”blood lead” OR TITLE_ABS:”bone lead” OR TITLE_ABS:”urinary lead” OR TITLE_ABS:”lead acetate” OR TITLE_ABS:”lead poisoning” OR TITLE_ABS:”lead toxicity” OR TITLE_ABS:”lead-induced” OR TITLE_ABS:”Pb exposure” OR TITLE_ABS:”Pb-induced” OR TITLE_ABS:”Pb toxicity” OR TITLE_ABS:plumbism) AND (TITLE_ABS:diabet* OR TITLE_ABS:glucose OR TITLE_ABS:”insulin resistance” OR TITLE_ABS:HOMA-IR OR TITLE_ABS:”triglyceride-glucose” OR TITLE_ABS:TyG OR TITLE_ABS:”metabolic syndrome” OR TITLE_ABS:”pancreatic beta cell” OR TITLE_ABS:”pancreatic β-cell” OR TITLE_ABS:gluconeogenesis) AND FIRST_PDATE:[1900-01-01 TO 2026-08-04].

### Appendix A.2. Redox/Mechanistic

PubMed/MEDLINE (1747 records): (“lead exposure”[tiab] OR “blood lead”[tiab] OR “bone lead”[tiab] OR “urinary lead”[tiab] OR “lead acetate”[tiab] OR “lead poisoning”[tiab] OR “lead toxicity”[tiab] OR lead-induced[tiab] OR “Pb exposure”[tiab] OR Pb-induced[tiab] OR “Pb toxicity”[tiab] OR plumbism[tiab]) AND (Nrf2[tiab] OR NFE2L2[tiab] OR Keap1[tiab] OR thioredoxin[tiab] OR TXNIP[tiab] OR TXNRD1[tiab] OR mitochondri*[tiab] OR “endoplasmic reticulum”[tiab] OR “gut microbiota”[tiab] OR “oxidative stress”[tiab]) AND (“1900/01/01”[dp]: “2026/08/04”[dp]).

Europe PMC (1724 records): (TITLE_ABS:”lead exposure” OR TITLE_ABS:”blood lead” OR TITLE_ABS:”bone lead” OR TITLE_ABS:”urinary lead” OR TITLE_ABS:”lead acetate” OR TITLE_ABS:”lead poisoning” OR TITLE_ABS:”lead toxicity” OR TITLE_ABS:”lead-induced” OR TITLE_ABS:”Pb exposure” OR TITLE_ABS:”Pb-induced” OR TITLE_ABS:”Pb toxicity” OR TITLE_ABS:plumbism) AND (TITLE_ABS:Nrf2 OR TITLE_ABS:NFE2L2 OR TITLE_ABS:Keap1 OR TITLE_ABS:thioredoxin OR TITLE_ABS:TXNIP OR TITLE_ABS:TXNRD1 OR TITLE_ABS:mitochondri* OR TITLE_ABS:”endoplasmic reticulum” OR TITLE_ABS:”gut microbiota” OR TITLE_ABS:”oxidative stress”) AND FIRST_PDATE:[1900-01-01 TO 2026-08-04].

### Appendix A.3. Acute Dose/LD_50_

PubMed/MEDLINE (51 records): (“lead acetate”[tiab] OR “Lead Compounds”[Mesh] OR “Lead Poisoning”[Mesh]) AND (LD50[tiab] OR “median lethal dose”[tiab] OR “acute toxicity”[tiab]) AND (“1900/01/01”[dp]: “2026/08/04”[dp]).

Europe PMC (165 records): (TITLE_ABS:”lead acetate” OR TITLE_ABS:”lead compound” OR TITLE_ABS:”lead poisoning”) AND (TITLE_ABS:LD50 OR TITLE_ABS:”median lethal dose” OR TITLE_ABS:”acute toxicity”) AND FIRST_PDATE:[1900-01-01 TO 2026-08-04].

### Appendix A.4. Supplementary Multidisciplinary Search

OpenAlex (25 targeted title-and-abstract queries; 2311 raw query hits): Twelve metabolic queries combined Pb exposure terms with diabetes, glucose, insulin resistance, metabolic syndrome, or gluconeogenesis (990 hits); ten mechanistic queries combined Pb exposure terms with Nrf2/NFE2L2, Keap1, thioredoxin/TXNIP, mitochondria, oxidative stress, gut microbiota, or endoplasmic reticulum (981 hits); and three acute-hazard queries combined lead acetate/compounds/poisoning with LD_50_, median lethal dose, or acute toxicity (340 hits). After within-family query consolidation, 712 metabolic, 878 mechanistic, and 339 acute-hazard records remained. The complete exact-query audit was retained by the authors.
